# Inosine triphosphate pyrophosphatase from *Trypanosoma brucei* cleanses cytosolic pools from deaminated nucleotides

**DOI:** 10.1038/s41598-022-10149-4

**Published:** 2022-04-18

**Authors:** Antonio E. Vidal, Miriam Yagüe-Capilla, Blanca Martínez-Arribas, Daniel García-Caballero, Luis M. Ruiz-Pérez, Dolores González-Pacanowska

**Affiliations:** Instituto de Parasitología y Biomedicina López-Neyra, Consejo Superior de Investigaciones Científicas, Parque Tecnológico de Ciencias de la Salud, Avenida del Conocimiento, 17, 18016 Armilla, Granada Spain

**Keywords:** Parasite biology, Hydrolases, DNA damage and repair, RNA metabolism

## Abstract

Inosine triphosphate pyrophosphatases (ITPases) are ubiquitous house-cleaning enzymes that specifically recognize deaminated purine nucleotides and catalyze their hydrolytic cleavage. In this work, we have characterized the *Trypanosoma brucei* ITPase ortholog (TbITPA). Recombinant TbITPA efficiently hydrolyzes (deoxy)ITP and XTP nucleotides into their respective monophosphate form. Immunolocalization analysis performed in bloodstream forms suggests that the primary role of TbITPA is the exclusion of deaminated purines from the cytosolic nucleoside triphosphate pools. Even though *ITPA*-knockout bloodstream parasites are viable, they are more sensitive to inhibition of IMP dehydrogenase with mycophenolic acid, likely due to an expansion of IMP, the ITP precursor. On the other hand, TbITPA can also hydrolyze the activated form of the antiviral ribavirin although in this case, the absence of ITPase activity in the cell confers protection against this nucleoside analog. This unexpected phenotype is dependant on purine availability and can be explained by the fact that ribavirin monophosphate, the reaction product generated by TbITPA, is a potent inhibitor of trypanosomal IMP dehydrogenase and GMP reductase. In summary, the present study constitutes the first report on a protozoan inosine triphosphate pyrophosphatase involved in the removal of harmful deaminated nucleotides from the cytosolic pool.

## Introduction

The cellular nucleotide pool can undergo unwanted chemical reactions such as oxidation, deamination or other modifications giving rise to noncanonical nucleoside triphosphates (NTPs). Deoxyuridine triphosphate (dUTP), 8-oxo-deoxyguanosine triphosphate (8-oxo-dGTP), (deoxy)xanthosine triphosphate ((d)XTP) or (deoxy)inosine triphosphate ((d)ITP) among others^1^, are noncanonical nucleotides that may either accumulate in nucleotide pools or become incorporated into DNA and RNA, leading to alterations of nucleic acid structure and genetic information. Their removal is therefore essential to maintain genetic integrity. This task is performed by the so-called ‘house-cleaning’ enzymes that include several families of specialized NTP pyrophosphates which catalyze the hydrolysis of noncanonical or damaged nucleosides triphosphate to their corresponding monophosphate form^2^.

Inosine triphosphate pyrophosphatases (ITPases, ITPAs) are house-cleaning enzymes that can be found in all three domains of life and whose function is to eliminate (d)ITP and XTP from nucleotide pools^2^. Several ITPases from different organisms have been structurally characterized; they all exhibit a high degree of similarity indicating that ITPases perform an important and evolutionarily conserved function^3–6^. Indeed, genetic studies in bacteria, yeast and mammalian models have found that deficiencies in ITPA activity cause severe genetic instability including high mutation rates, accumulation of noncanonical purines in nucleic acids and chromosomal damage^7–9^. Moreover, ITPase knockout mice show growth retardation, heart abnormalities, ataxia and die before weaning^10^. In humans, the vital role of ITPA is emphasized by the finding that certain homozygous null mutations in the *itpa* gene result in early lethal infantile encephalopathy or cardiomyopathy^11,12^.

*Trypanosoma brucei* is a protozoan parasite and the etiological agent of African Trypanosomiasis (HAT) also known as sleeping sickness in humans and as nagana disease in cattle. Once inside the mammalian host, the parasites invade the bloodstream and lymph system. Eventually, they cross the blood–brain barrier to initiate the second stage of the disease, which causes the characteristic sleep disorder and is ultimately fatal if left untreated. Most of the protozoan parasites rely on purine salvage, as none have the de novo synthesis pathway^13^. This metabolic limitation has led to consider the trypanosomal purine salvage pathway as a promising chemotherapeutic target^14^. In *T. brucei*, the salvage pathway involves an extense family of nucleoside and nucleobase transporters with different substrate affinities and expression profiles along the trypanosome life cycle^15^, nucleoside hydrolases^16^, 6-oxopurine PRTases^17^, adenine PRTases^18^, an adenosine kinase^19^ as well as several enzymes responsible for nucleotide interconversion such as adenylosuccinate lyase (ADSL), adenylosuccinate synthetase (ADSS)^20^, GMP synthase (GMPS)^21^, GMP reductase^22^ and inosine-5-monophosphate dehydrogenase (IMPDH)^23^. Despite this apparent metabolic redundancy, some of the enzymes of the salvage pathway are essential which can be explained by the limited availability of purine precursors in the mammalian host environment (blood serum, cerebrospinal fluid, etc.) or by the toxic accumulation of metabolic intermediates^21,24^. Among the potentially toxic intermediates produced as a by-products of purine metabolism are deaminated purine nucleotides that, if not removed by the ITPase, can be deleterious for the cell. ITP and XTP can arise either by spontaneous or induced (e.g., by oxidative stress or inflammation) deamination of adenine or guanine nucleotides^25^ or due to the unwanted phosphorylation of two early intermediates of the purine synthesis pathway: inosine monophosphate (IMP) and xanthosine monophosphate (XMP). Accordingly, imbalances in purine metabolism caused by genetic defects in the enzymes that regulate the pools of IMP and XMP increase the burden of deaminated nucleobases in RNA, especially in ITPase-deficient cells^7^.

Here we report the characterization of the *T. brucei* ortholog of ITPase (TbITPA), the enzyme responsible for the conversion of noncanonical (d)ITP and XTP nucleotides into their respective monophosphate forms and pyrophosphate. The aim of the present study was to elucidate the role for this protein in the cleansing of purine pools in the parasite. To gain further insight into this NTP pyrophosphatase, we performed a kinetic analysis of the recombinant protein, determined its intracellular localization and evaluated the phenotype of *itpa*-deficient *T. brucei* blood-stage cells. The data presented suggest that TbITPA has a primary role in excluding noncanonical purines from cytosolic nucleoside triphosphate pools, including the activated form of the antiviral ribavirin. However, the presence of deaminated purines does not appear to be sufficiently relevant to induce cell toxicity under normal in vitro growth conditions unless IMP pools are expanded through IMPDH inhibition. Overall, ITPA-deficient cells have proved to be a valuable tool to better understand purine metabolism in the parasite.

## Results

### Enzymatic analysis of the inosine triphosphate pyrophosphatase from *Trypanosoma brucei*

**Figure 1:**
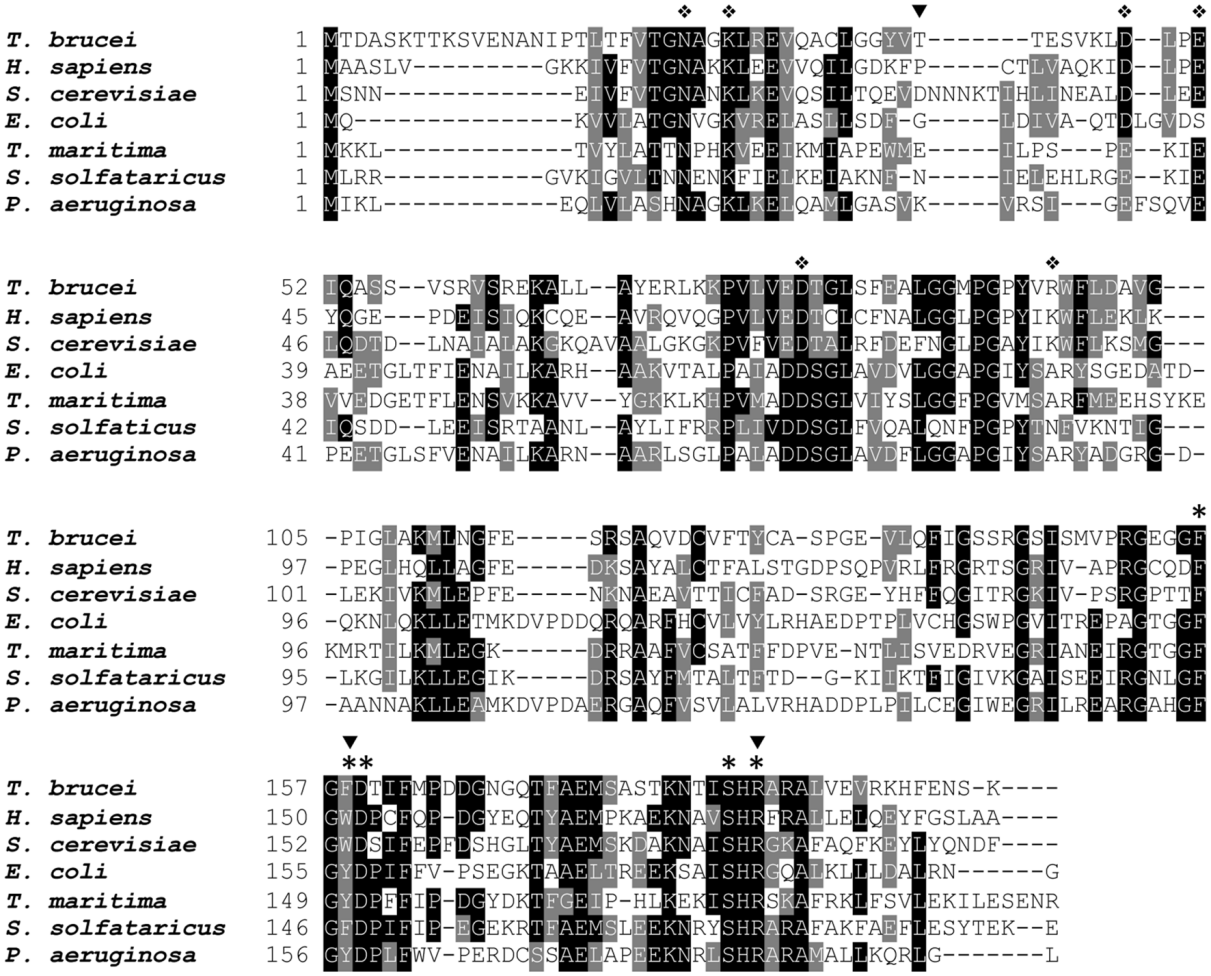
Sequence alignment of TbITPA with other six characterized orthologs from prokaryotic and eukaryotic organisms. Multiple sequence alignment was generated using T-Coffee and the final format was obtained using Boxshade. The amino acids involved in substrate selectivity (*), catalysis (❖) as well as those associated with clinical phenotypes (▼) are indicated. Protein sequences were retrieved from NCBI RefSeq: *T. brucei*, *Trypanosoma brucei* (XP_823221.1); *H. sapiens*, *Homo sapiens* (NP_258412.1); *S. cerevisiae*, *Saccharomyces cerevisiae* (NP_012603.1); *E. coli*, *Escherichia coli* (NP_417429.1); *T. maritima*, *Thermotoga maritima* (NP_227974.1); *S. solfataricus, Sulfolobus solfataricus* (WP_009988745.1); *P. aeruginosa, Pseudomonas aeruginosa* (NP_249078.1).

Human ITPA protein sequence was used to query the *Trypanosoma brucei brucei* TREU927 genome database in order to find an ortholog sequence. As a result, we identified a protein with high homology (identity = 46%; E = 1 × 10^–43^; bits = 146) annotated as a putative non-canonical purine NTP pyrophosphatase (Tb927.10.9990) of 201 amino acids and a calculated mass of 21.6 kDa. The *T. brucei* ITPA protein (TbITPA) sequence was aligned with other six characterized orthologs from prokaryotic and eukaryotic organisms (Fig. 1). Most of the amino acids identified as crucial for catalysis and substrate discrimination in human ITPA (i.e., Glu44, Asp72, Arg178, F149, etc.) are conserved in TbITPA^26^. In addition, TbITPA exhibits high similarity with *Leishmania major* (47% identity) and *T. cruzi* ITPases (58% identity), and critial residues are all present (Fig. S1). It is of interest to note that residue Pro32 in human ITPA is substituted by Thr41 in the TbITPA enzyme. A similar amino acid variant that results in decreased enzyme activity^27,28^ has been found as a consequence of a frequent human genetic polymorphism (94C>A)^29^.

**Figure 2:**
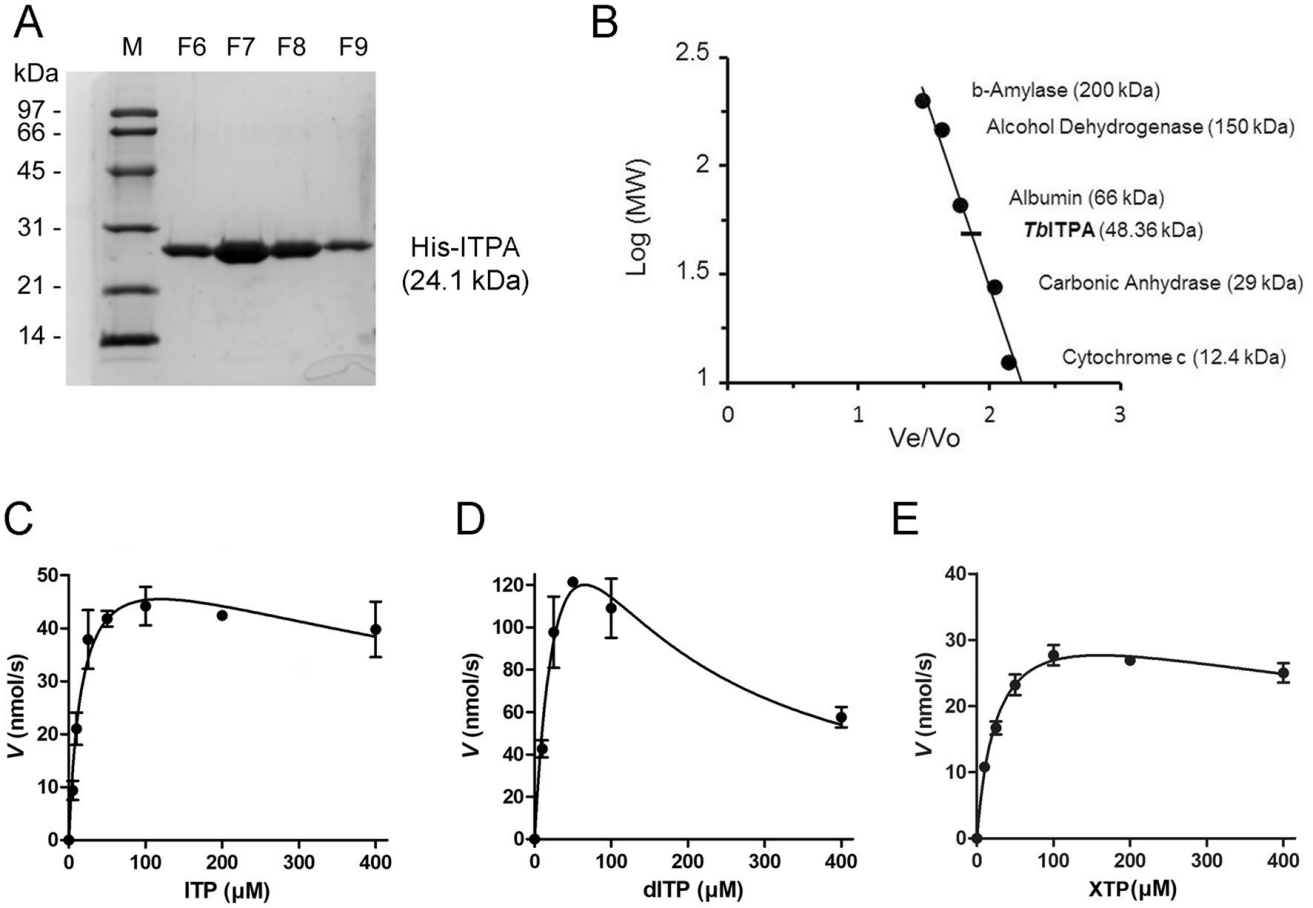
TbITPA exhibits NTP pyrophosphatase activity over non-canonical NTP substrates. (**A**) Purified protein was separated on SDS-PAGE gel and stained with Coomassie. M, molecular weight markers; F6-F9, fractions containing purified TbITPA were obtained after affinity and anion exchange chromatography. (**B**) A sample of purified TbITPA protein was applied to a Superdex 200 10/300 GL column (GE Healthcare) in 50 mM phosphate, 0.15 M NaCl, pH 7.2 and eluted at 0.5 mL/min in the same buffer. The molecular weight (MW) determination is extrapolated by comparing the Ve/Vo ratio for TbITPA to the Ve/Vo of protein standards of known molecular weight, Ve = Elution volume of the protein; Vo = Void volume of the column. (**C**) Substrate saturation curves obtained for ITP, (**D**) dITP and (**E**) XTP. Each data point is the mean (± SD) of at least three independent determinations. Reactions were performed and analyzed as described in Materials and Methods. The data were fitted to a non-linear kinetic model for substrate inhibition and analyzed with GraphPad Prism 5 software.

In order to determine the catalytic activity of TbITPA, its coding sequence was cloned in the *E. coli* vector pET28a that allows expression of the protein fused to a hexahistidine peptide at the N-terminus. Histidine-tagged recombinant protein (24 kDa) was purified by metal-affinity and subsequently by anion exchange chromatography to near homogeneity (Fig. 2A, Fig. S2). Gel filtration analysis revealed that TbITPA elutes with a calculated molecular weight of 48 kDa, as expected for the corresponding homodimeric form (Fig. 2B).

It is well established that ITPase (E.C. 3.6.1.19) proteins convert non-canonical purine (d)NTPs, such as (deoxy)inosine 5′-triphosphate, into their corresponding nucleoside monophosphate, releasing pyrophosphate (PPi). To validate TbITPA as a *bona fide* inosine triphosphate pyrophosphatase (ITPase), the enzyme was incubated in the presence of deaminated purine nucleotides and reaction products were analyzed by HPLC. Reaction conditions were empirically determined using ITP as substrate. At pH 7.5, ITP hydrolysis was found to be optimal at 5 mM MgCl_2_ in the absence of salts (NaCl). Substrate saturation curves and kinetic parameters for ITP, dITP and XTP were obtained (Fig. 2C–E and Table 1). Under the established reaction conditions, TbITPA can efficiently hydrolyze ITP (*k*_cat_ = 59 ± 5 s^−1^; *K*_m_ = 17 ± 3 μM) and dITP (*k*_cat_ = 280 ± 74 s^−1^; *K*_m_ = 44 ± 18 μM) to (d)IMP and XTP to XMP (*k*_cat_ = 37 ± 2 s^−1^; *K*_m_ = 27 ± 4 μM) but no activity was detected over the canonical nucleotides ATP, GTP, dATP and dGTP. The concentration *vs* rate data reveals substrate inhibition properties at high concentrations. A similar feature has been reported for ITPases from other organisms although its biological relevance is not completely understood^30,31^.

**Table 1:** Kinetic parameters for TbITPA.

	k_cat_ (s^−1^)	K_m_ (μM)	k_cat_/K_m_ (μM)	K_i_ (μM)^a^
ITP	59 ± 5	17 ± 3	3.5 ± 1.7	824 ± 275
dITP	280 ± 74	44 ± 18	6.4 ± 4	98 ± 42
XTP	37 ± 2	27 ± 4	1.4 ± 0.5	951 ± 220

^a^Substrate inhibition constant.

### TbITPA is not essential in *T. brucei* bloodstream forms (BSF)

**Figure 3:**
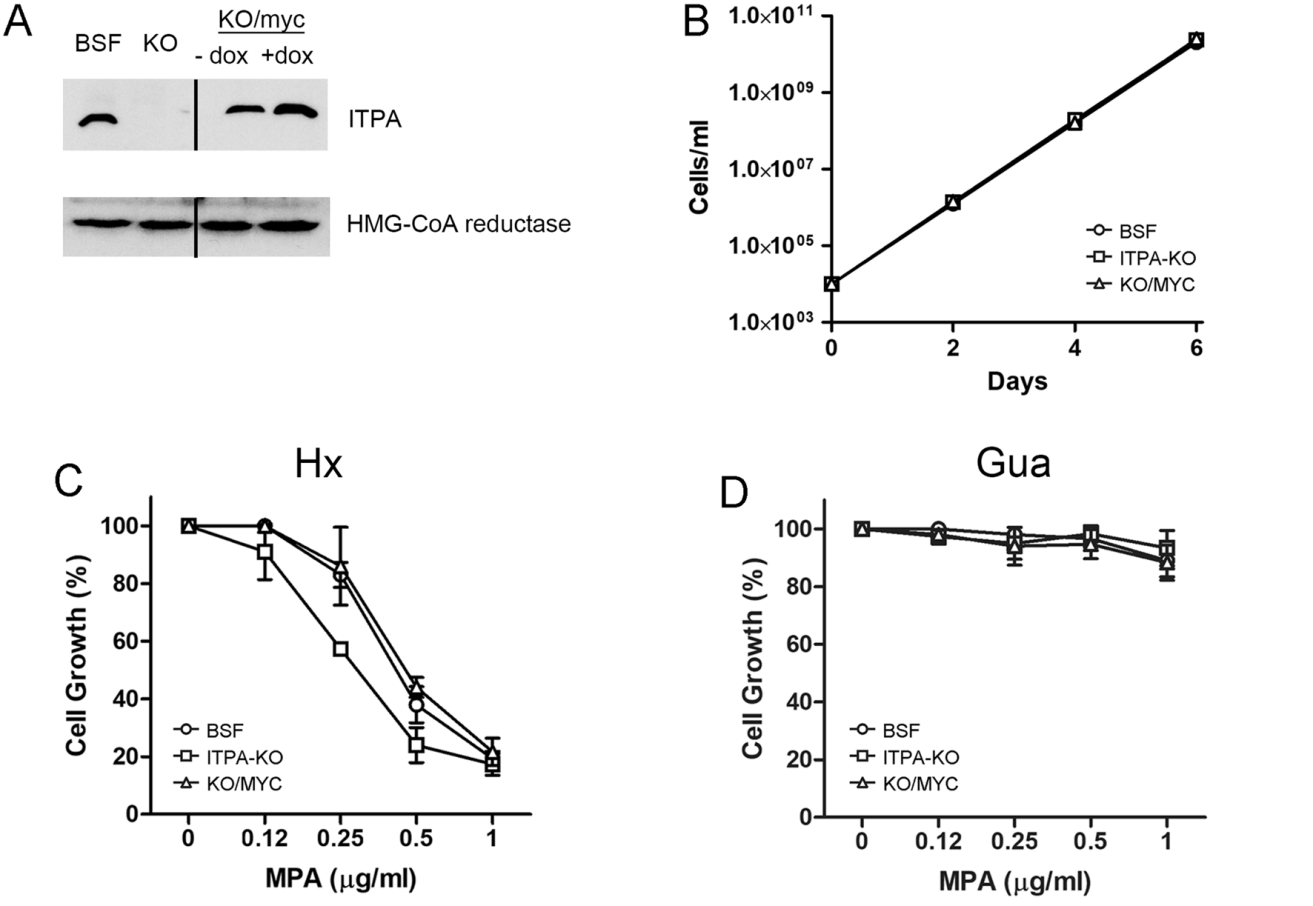
Effect of ITPA inactivation in bloodstream *T. brucei* cells. (**A**) Western blot showing ITPA protein levels in whole cell extracts from 5 × 10^6^ parasites. ITPA was detected with the anti-ITPA polyclonal antibodies. Cell lines analyzed include: wild-type bloodstream form (BSF), ITPA-KO cells (KO) or ITPA-KO cells harboring an ectopic inducible copy of a myc-ITPA fusion gene (KO/myc) in the absence (− dox) or presence (+ dox) of the inductor. As loading control, HMG-CoA reductase protein was measured in a separate gel with anti-HMG-CoA reductase polyclonal antibodies. Images were cropped and aligned for easier comparison. (**B**) Cumulative cell growth over a six-day period of trypanosomes cultured in standard HMI-9 medium in vitro. KO/myc cell line was grown in the presence of the inductor. Data represent the mean for three independent biological replicates. (**C**, **D**) Log-phase parasites at 10^5^ cells mL^−1^ were exposed to increasing concentrations of mycophenolic acid (MPA) for 24 h at 37 °C in HMI-9 medium containing (**C**) hypoxanthine (1 mM) or (**D**) modified HMI-9 medium containing guanine (100 µM) as the sole purine source. Proliferation was calculated relative to cell growth in the absence of MPA for each cell line. Experiments were performed at least three times, values are the mean (± SD). Cell lines are represented with the following symbols: BSF (circle), ITPA-KO (square), KO/myc-ITPA in the presence of 1 μg mL^−1^ of doxycycline (triangle).

To evaluate the essentiality of TbITPA in *T. brucei*, we proceeded to generate an *itpa* null cell line by double allele replacement in BSF parasites (Lister 427 single marker cells). Two DNA constructs were made containing the blasticidin S deaminase (BSD) and hygromycin phosphotransferase (HYG) resistance markers flanked by approximately 600 base pairs of the *ITPA* 5′-UTR and 3′-UTR sequences. Linear targeting fragments were transfected sequentially into BSF cells and after each transfection, recombinant clones were selected with the appropriate drug. Only the double knockout clone does not retain any copy of the *ITPA* gene as shown by the absence of PCR product in the PCR assay with *ITPA*-specific primers (Fig. S3A,B). Correct integration of the BSD and HYG markers in the double knockout cells was verified by PCR analysis using primers that hybridize either upstream or downstream of the inactivation cassettes in combination with marker-specific primers (Fig. S3C,D). Successful inactivation of the *itpa* gene was further confirmed by Western blot (Fig. 3A, Fig. S4), thus providing direct evidence that TbITPA is not essential for trypanosome viability under standard in vitro growth conditions.

The ITPA-KO cell line was then transfected with a DNA construct containing the *itpa* gene fused to a c-myc epitope sequence at the 5′-end that allow gene integration at rDNA regions and selection by puromycin. The resulting cell line provides an ITPA-KO background that expresses the ITPase from a different locus (KO/myc). Even though the fusion mycITPA construct was flanked by sequences that allow regulation of expression by doxycycline, all of the clones selected expressed mycITPA constitutively even in the absence of the inductor (Fig. 3A).

Figure 3B shows the cumulative growth of the various cell lines over a 6-day period. Parasites lacking TbITPA exhibit a similar proliferation rate than parental BSF cells or KO cells expressing myc-TbITPA. The observation that TbITPA is non-essential agrees with results obtained in a genome-wide RNAi screen showing that knockdown of ITPA by RNAi does not cause significant loss of fitness in BSF or PCF (procyclic forms) parasites^32^.

### ITPA deficiency sensitizes trypanosomes to inhibition of IMP dehydrogenase

IMP dehydrogenase (IMPDH) catalyzes the NAD^+^-dependent oxidation of IMP to XMP and is a rate-limiting step in the synthesis of guanine nucleotides. Inactivation of IMPDH has been associated with increased accumulation of inosine in RNA and DNA in bacteria^7^. This is consistent with an expansion of the IMP pool and excess IMP becoming a substrate for kinases and ribonucleotide reductase to generate expanded (d)ITP pools. This observation prompted us to investigate the impact of IMPDH inactivation on cells lacking ITPA and therefore unable to prevent (d)ITP incorporation into RNA/DNA. It has been reported that *T. brucei* IMPDH activity is efficiently inhibited by several molecules including mycophenolic acid (MPA), ribavirin 5′-monophosphate, and mizoribine 5′-monophosphate with *K*_i_ values of 21 nM, 3.2 μM and 3.3 nM, respectively^23^. Here, we have used MPA to inhibit TbIMPDH activity in *T. brucei* cell lines expressing or not ITPase activity. The proliferation assays were performed using HMI-9 as cell culture medium, which contains hypoxanthine as the sole purine source. Once in the cell, hypoxanthine can be converted into IMP by one of the three 6-oxopurine phosphoribosyltransferases (6-oxoPRTases) present in *T. brucei*^17^. Under such strict hypoxanthine regime, IMP is the first and only intermediate generated by the purine salvage pathway and this makes trypanosomes fully dependent on IMPDH to initiate the conversion of IMP into GTP. Hence, it is expected that inhibition of IMPDH by MPA deprives the cell of guanine nucleotides. Indeed, exposure of parental BSF cells to MPA resulted in a significant growth inhibition, with similar levels of toxicity to those previously reported^33^ (Fig. 3C). Notably, trypanosome cells lacking the two *itpa* alleles were even more sensitive to MPA than BSF cells but this additional growth defect was reverted by the ectopic expression of mycITPA (Fig. 3C). This observation supports the notion that the toxic effect of MPA is partially due to the accumulation of ITP in the cellular nucleotide pool.

In line with this assumption, growth impairment caused by IMPDH inhibition is no longer observed when cells are supplemented with guanine instead of hypoxanthine (Fig. 3D). In the presence of guanine, cells do not require IMPDH for the synthesis of GMP, which is now directly provided by the activity of 6-oxopurine PRTases. Likewise, treatment with increasing concentrations of MPA in the presence of guanine did not have a significant impact on cell proliferation of ITPA-deficient cells, most probably due to low levels of IMP and ITP when guanine is the purine source.

### Trypanosomal ITPA mainly localizes in the cytoplasm

**Figure 4:**
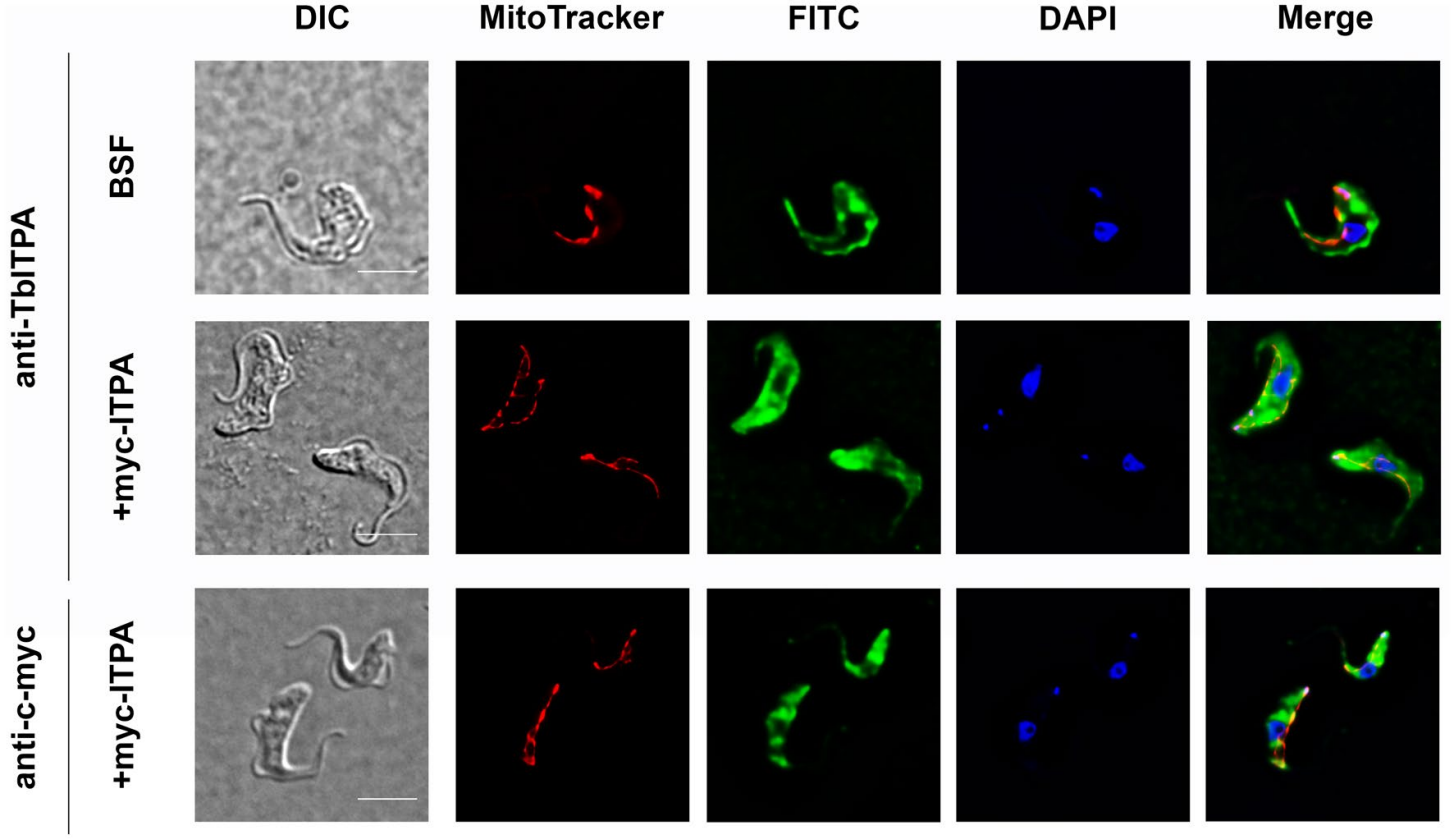
Subcellular localization of ITPA in bloodstream *T*. *brucei* cells. Immunofluorescence microscopy analysis was performed to establish the subcellular localization of ITPA in parental bloodstream *T. brucei* cells (BSF) (upper row) and mycTbITPA-overexpressing cells (+ myc-ITPA) (middle and lower row). Nuclear and kinetoplast DNA were stained with DAPI. The mitochondrion was visualized with MitoTracker Red CMXRos and ITPA was detected with anti-ITPA and Alexa Fluor 488 goat anti-rabbit as secondary antibody (upper and middle row) or anti-myc and Alexa Fluor 488 goat anti-mouse as secondary antibody. Images were collected with an Olympus microscope and Cell R IX81 software.

Mammalian ITPase has been mostly detected in the cytoplasm and to a lesser extent in the nucleus of different cell types^34^. Here, our aim was to obtain the first experimental evidence of the subcellular localization in *T. brucei* bloodstream parasites of ITPA under endogenous expression conditions. To this end, specific rabbit polyclonal antibodies against TbITPA were raised using soluble recombinant His-TbITPA as antigen. To ensure higher specificity, antibodies were purified from blood serum by affinity chromatography against the same pure recombinant protein. As shown in Fig. 4 (upper row), endogenous TbITPA protein was clearly detected by immunofluorescence microscopy in the cytoplasm of bloodstream *T. brucei* cells. The presence of the protein in the nucleus was carefully analyzed by determining Pearson’s correlation and Manders’ overlap coefficients. Manders’ coefficient indicates that only 10% of the DAPI signal overlaps with TbITPA staining (M2 = 0.10 ± 0.06). On the other hand, the Pearson’s correlation coefficient was not significant (0.17 ± 0.07). Considering these parameters, it is most probable that the major localization of ITPA is in the cytoplasm. A similar subcellular distribution was seen in a *T. brucei* cell line that overexpresses a C-terminal Myc-tagged version of TbITPA using both, the polyclonal anti-TbITPA (Fig. 4, middle row) and a monoclonal anti-myc antibody (Fig. 4, lower row).

### ITPase levels modulate ribavirin metabolism and toxicity in trypanosomes

Ribavirin (RBV) is an antiviral purine nucleoside analog with in vitro activity against a broad spectrum of RNA and DNA viruses. It is currently used in the treatment of Hepatitis C as part of drug-combination therapies. A recent work has shown that ribavirin triphosphate (RTP), the bioactivated form of RBV, is hydrolyzed by human ITPA with similar efficiency as its natural substrate ITP^35^. To further explore the substrate specificity of the trypanosomal enzyme, TbITPA was assayed by HPLC for activity against RTP under the same reaction conditions used with ITP. Kinetic analysis confirmed that, similarly to the human enzyme, TbITPA can hydrolyze RTP (*k*_cat_ = 100 ± 14 s^−1^; *K*_m_ = 75 ± 24 μM) with a specificity constant very similar to that obtained for ITP (*k*_cat_/*K*_m_ = 1.3 ± 0.6 s^−1^ μM^−1^) (Fig. S5).

**Figure 5:**
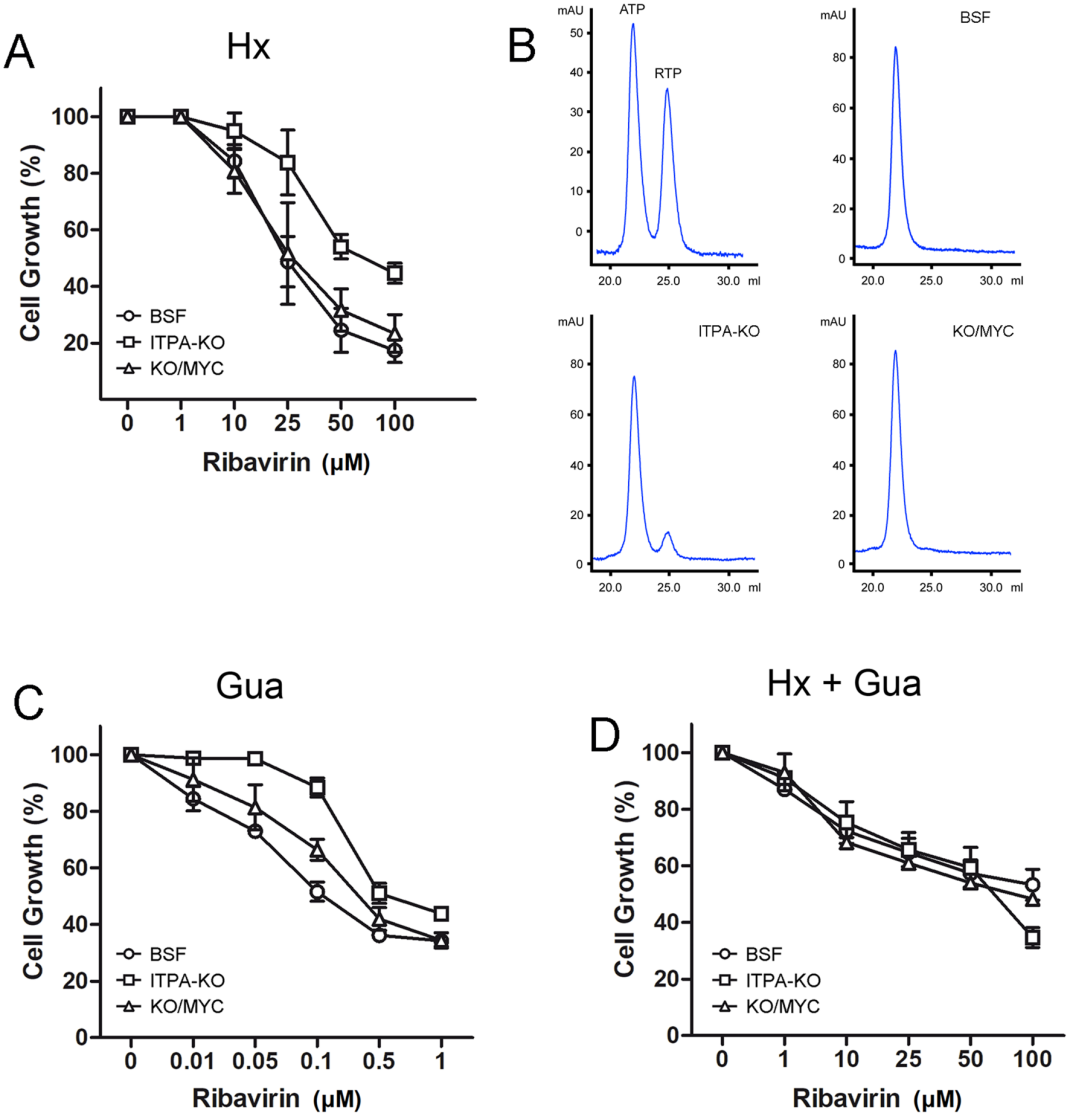
Antiproliferative effect of the antiviral ribavirin on trypanosomes is modulated by ITPA and the purines source. (**A**) Log-phase parasites at 10^5^ cells mL^−1^ were exposed to increasing concentrations of ribavirin (RBV) for 24 h at 37 °C in HMI-9 medium containing hypoxanthine (1 mM) as the sole purine source. (**B**) Intracellular detection of RTP by anion exchange chromatography. Top left panel represents the elution standards for ATP and RTP (**C**) Ribavirin sensitivity assay was performed in modified HMI-9 medium containing guanine (100 μM) as the sole purine source. (**D**) Ribavirin sensitivity assay was performed in modified HMI-9 medium containing guanine (100 μM) and hypoxanthine (100 μM). Values are the mean (± SD) of at least three independent experiments. Cell lines are represented with the following symbols: BSF (circle), ITPA-KO (square), KO/myc-ITPA in the presence of 1 μg mL^−1^ of doxycycline (triangle).

This observation led us to investigate whether TbITPA may confer cellular protection against RBV by removing the activated (triphosphate) form from the nucleotide pool. Thus, trypanosome cell lines with or without ITPase activity were exposed to increasing concentrations of RBV for 24 h before the effect on proliferation was evaluated (Fig. 5A). Unexpectedly, knockout cells deficient in ITPase activity were significantly less sensitive to RBV than parental or knockout cells expressing TbITPA from a different locus. Intracellular RTP levels do not seem to correlate with defective proliferation since the analysis by HPLC (high-performance liquid chromatography) of the RTP content in trypanosomes exposed to 100 μM ribavirin showed detectable levels of the activated form in ITPA-knockout cells but not in BSF or KO cells expressing myc-TbITPA where RTP is efficiently metabolized (Fig. 5B). An alternate explanation for the observed phenotype is that RTP hydrolysis by TbITPA generates ribavirin 5′-monophosphate (RMP), a potent TbIMPDH inhibitor^23^, which could be responsible for the toxic effect of RBV. Inhibition of IMPDH may lead to a block of GTP synthesis unless cells are provided with a direct source for guanine nucleotides. However, RMP also inhibits TbGMPR (GMP reductase) activity with a *K*_i_ value similar to that observed for the inhibition of TbIMPDH^22^. GMPR catalyzes the reductive deamination of GMP to IMP in the presence of NADPH. Therfore, parasite treatment with RBV can also induce a block in the conversion of guanine nucleotides into adenine nucleotides. It is therefore expected that trypanosomes grown in medium supplemented with only guanine as purine source and exposed to RMP will be deficient in the synthesis of adenine nucleotides; only supplementation with both nucleobases, hypoxanthine and guanine, would avoid the need for IMPDH and GMPR activities*.* To reevaluate the impact of ITPA in RBV sensitivity, cells were this time incubated in the presence of guanine (100 μM) (Fig. 5C) or both guanine (100 μM) and hypoxanthine (100 μM) (Fig. 5D). In the presence of guanine as the sole purine in the medium, all trypanosome lines were much more sensitive to RBV probably due to an increased uptake in the absence of hypoxanthine. Indeed, an upregulation of the nucleoside P1 transporter, responsible for the uptake of ribavirin^36^, has been documented in *T. brucei* in response to purine stress^37^. Comparing cell lines, the degree of sensitivity to RBV was similar to the observed in a medium with only hypoxanthine; cells lacking ITPA were more resistant to RBV suggesting that accumulation of the RMP intermediate is still toxic in a medium with only guanine. In contrast, this effect disappeared when cells were grown at equimolar concentrations of guanine and hypoxanthine. When both nucleobases are made available, ITPase-deficient and -proficient cells are inhibited by RBV in a similar manner, except at high concentrations where RTP hydrolysis may play a protective role. Overall, these observations show a clear role for TbITPA in the metabolic fate of ribavirin and reveal a relationship between its toxic effect and purine availability.

## Discussion

ITPases are a group of ‘house-cleaning’ enzymes that catalyze the pyrophosphohydrolysis of deaminated purine nucleotides thus reducing their harmful incorporation into nucleic acids^5,30,31^. A putative ITPA ortholog from *T. brucei* was retrieved by BLAST search of the TriTrypDB database. Our data provide experimental evidence that trypanosomal ITPase is stably expressed and accumulates preferentially in the cytoplasm of *T. brucei* bloodstream cells. Moreover, the recombinant protein exhibits NTP pyrophosphatase activity over the deaminated nucleotides (d)ITP and XTP. Overall, enzymatic properties of recombinant TbITPA were shown to be very similar to those previously described for human ITPase, behaving as a homodimer under physiological conditions and exhibiting substrate inhibition^30^.

The ITPA homolog from *T. brucei* shares 46% of amino acid sequence identity with human ITPA including those residues catalytically relevant. However, there is a remarkable difference in the amino acid sequence with respect to the human enzyme involving the replacement of residue Pro32 by threonine (Thr41) in *T. brucei*. This replacement is present in several reported *T. brucei* ITPA sequences. The referred amino acid substitution in human ITPA has been shown to arise from a genetic missense polymorphism, 94C>A (Pro32 to Thr) which is frequently detected in Asians (up to 19%), Caucasians and Africans (6–7%)^29^. This protein variant has been associated with a severe ITPase deficiency in homozygote 94C>A mutants even though purified recombinant mutant enzyme still retains significant activity^38,39^. Rather, mutation 94C>A seems to promote defects in pre-mRNA splicing and increased protein instability by destabilizing the hydrophobic core of the protein^28,40^. As for TbITPA, the recombinant enzyme is fully active in vitro and maintains the dimeric conformation described for human ITPA. In addition, mis-splicing events can be ruled out in this case since most of *T. brucei* genes, including the *itpa* gene, lack introns. Moreover, western blot and immunofluorescence analysis shows that the enzyme is expressed and stable in its cellular environment. It is therefore likely that additional residues or factors in the architecture of the hydrophobic core help to maintain the stability of TbITPA.

Kinetic analysis revealed similar specificity constants for TbITPA over ITP, XTP and dITP. The *K*_m_ values of TbITPA for ITP and XTP (17 and 27 μM, respectively) are well below the reported intracellular concentrations of their corresponding canonical NTPs (1600 and 380 μM for ATP and GTP respectively)^41^ suggesting that the enzyme efficiently contributes to minimising the expansion of the pool of non-canonical purine ribonucleoside triphosphates. On the other hand, the higher *K*_m_ value for dITP (44 μM) may indicate that it is a less relevant substrate considering the low intracellular concentration of dATP (10 μM)^41^. The cytosolic location of TbITPA supports a house-cleaning function for TbITPA of the cytosolic NTP pools that are going to be used during transcription although its role in minimizing dNTP pools cannot be discarded. A previous study of ITPA-knockout mice led to the same conclusion; although inosine ribonucleotides accumulated significantly in both the nucleotide pool and RNA, inosine was also found in DNA^10^.

*Trypanosoma brucei* must cope with the nitrosative/oxidative burst from the host immune system, which includes several reactive nitrogen and oxygen species released by phagocytes in the early steps of an infection. Upon exposure to these genotoxic agents, the nucleotide pool is prone to undergo base deaminations via hydrolytic and nitrosative reactions^25^, producing aberrant non-canonical ribonucleotides like ITP and XTP. If not eliminated from the pool, they could be erroneously incorporated by RNA polymerases during transcription. The substitution of adenine for hypoxanthine and of guanine for xanthine in messenger, transfer or ribosomal RNAs could affect cell viability by interfering with the structure of functional RNAs and protein translation^42,43^. Additionally, other biological functions may be negatively affected by deaminated nucleotides; ITP might compete with ATP or GTP and inhibit certain biochemical processes as reported for microtubule polymerization^44^.

**Figure 6:**
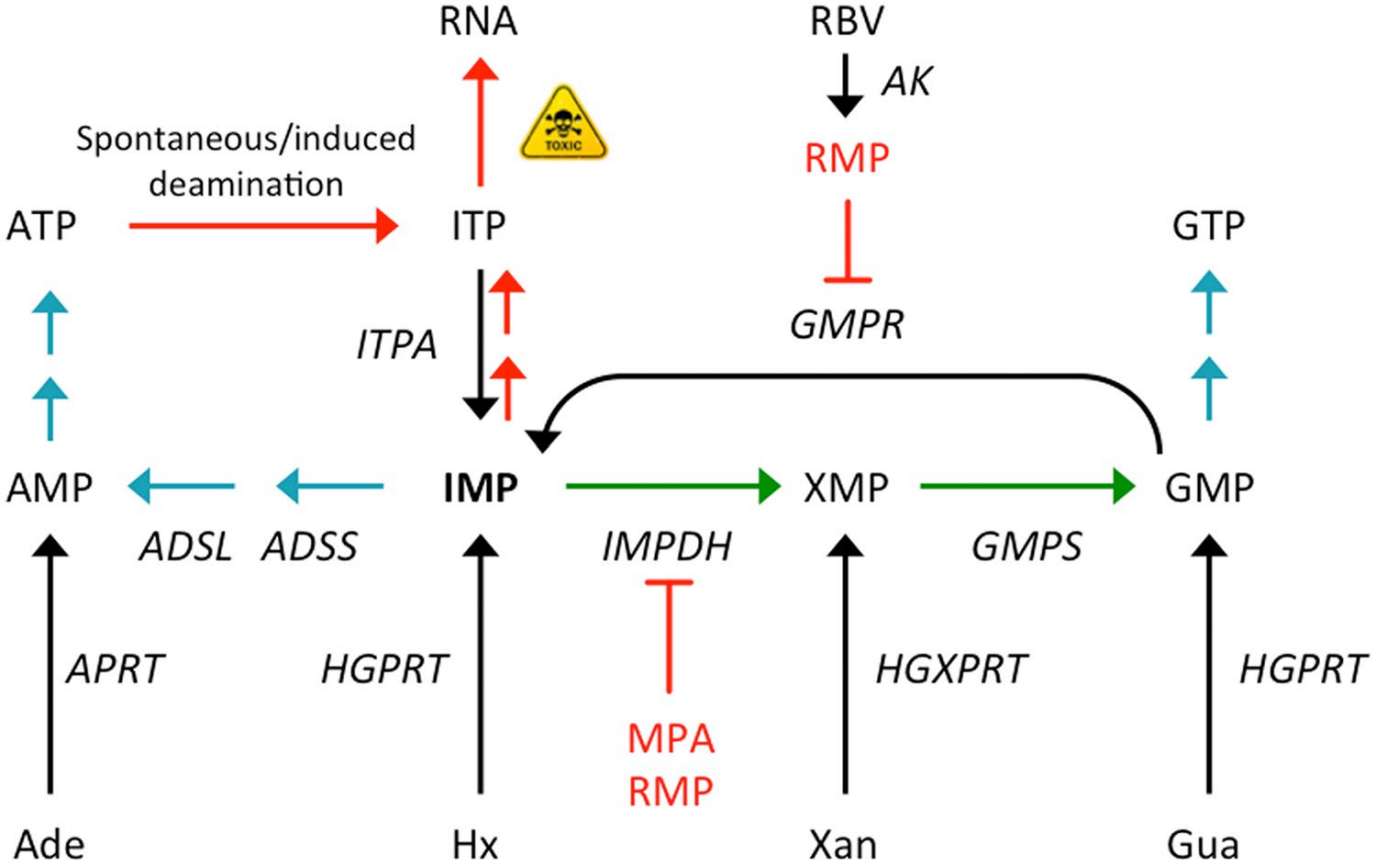
Role of ITPA in purine metabolism in *T. brucei*. Simplified schematic representation of the purine salvage and interconversion enzymes identified in *T. brucei*. ITP can arise from ATP by spontaneous or induced deamination or from consecutive phosphorylation of IMP. Enzymatic activities are depicted as follows: ITPA, inosine triphosphate pyrophosphatase; APRT, adenine phosphoribosyltransferase; IMPDH, inosine monophosphate dehydrogenase; GMPS, guanine monophosphate synthase; GMPR, guanosine monophosphate reductase; ADSS, adenylosuccinate synthetase; ADSL, adenylosuccinate lyase; HGPRT, hypoxanthine–guanine phosphoribosyltransferase; HGXPRT, hypoxanthine–guanine–xanthine phosphoribosyltransferase; AK, adenosine kinase; Ade, adenine; Hx, hypoxanthine; Xan, xanthine; Gua, guanine; RBV, ribavirin. Specific inhibitors studied in this work are also shown: MPA, mycophenolic acid; RMP, ribavirin monophosphate.

Unlike its essential role in mammalian cells^10–12^, ITPA activity seems to be dispensable in *T. brucei* bloodstream forms under standard culture conditions. In trypanosomes, the accumulation of deaminated purine nucleotides does not appear sufficient to impair growth and viability. It is likely that ATP deamination is not significant under in vitro growth conditions and the IMP pool is effectively channeled to AMP and GMP synthesis before being converted into ITP by phosphorylation (Fig. 6). A viable phenotype was also displayed by *E. coli rdgB* and *S. cerevisiae HAM1* mutants where the ITPase activity is not essential for survival^45,46^. The lack of ITPase however, provoked the accumulation of (deoxy)inosine in RNA and DNA in these mutants especially in combination with defects in IMP dehydrogenase or adenylosuccinate synthetase, activities that regulate the pool of IMP^7^. Similarly, we have found that ITPA-depleted trypanosomes show increased susceptibility to IMPDH inhibition by mycophenolic acid in a medium containing hypoxanthine as the sole purine source, conditions that promote the expansion of the IMP pool. It remains to be determined whether the loss of ITPase activity is causally associated with decreased infectivity and virulence in vivo where oxidative-nitrosative stress-induced deaminations are likely exacerbated.

Beyond its physiological function, a pharmacogenetic relationship between ITPA and the toxic side-effects of the antiviral ribavirin has been well established. Fellay et al. first demonstrated that genetic variants leading to inosine triphosphatase deficiency protect against haemolytic anaemia in chronic hepatitis C patients treated with ribavirin (RBV)^47^. The mechanism underlying this protective effect is still under debate but different hypotheses suggest that the modulation of the NTP pools plays a role^48^. While it is clear that erythrocytes and trypanosomes differ in many aspects of purine metabolism including purine availability, their respective enzymatic repertoires and the size and proportion of their NTP pools, we found an evident parallelism between the resistance to RBV observed in ITPA-KO trypanosomes and the protective effect of human ITPA deficient variants against RBV-induced hemolysis. The use of a eukaryotic unicellular model easy to genetically and chemically manipulate such as the trypanosome has allowed us valuable insights into the impact of ribavirin in purine nucleotide synthesis. Early studies showed that ribavirin is phosphorylated by human adenosine kinase^49^. *T. brucei* is also endowed with an adenosine kinase with capacity to salvage adenosine^19^ which may also be involved in the conversion of ribavirin into ribavirin monophosphate (RMP) (Fig. 6). On the other hand, we have shown that TbITPA efficiently hydrolyzes ribavirin triphosphate (RTP) back to its monophosphate form. Hence, it is expected that in cells treated with RBV, the presence of ITPase activity promotes the expansion of a RMP pool, an intermediate that has been shown to inhibit *T. brucei* IMPDH and GMPR enzymes with *K*_i_ values in the low micromolar range (Fig. 6)^22,23^. Therefore, the accumulation of RMP may result in further toxicity for the cell when purine nucleotide synthesis depends on only one of these activities. This deleterious effect can be bypassed thanks to the ability of *T. brucei* to uptake and salvage different purine bases^17^. Thus, when trypanosomes have various purine sources at their disposal such as hypoxanthine and guanine, they do not require the interconversion enzymes IMPDH or GMPR to produce GMP and IMP respectively (Fig. 6). In summary, in trypanosomes the toxicity of RBV that is associated with ITPA depends on purine availability and relies on the accumulation of the monophosphate form (RMP). We believe these findings may also help to explain the pharmacogenetic relationship between ITPA and the toxic side-effects of ribavirin in chronic hepatitis C patients. Moreover, ITPA needs to be considered as a factor modulating the action of inosine or adenosine analogs with antitrypanosomal activity such as a recently discovered series of *O*-alkylated 7-deazainosine derivatives^50^.

The experiments presented in this work show that ITPA is a genuine inosine triphosphate pyrophosphatase which likely plays a role in the removal of the potentially harmful ITP and dITP from the cytosolic nucleotide pool. In trypanosomes, ITP and dITP could primarily arise from ATP and dATP deamination under the oxidative and nitrosative stress generated by the immune defenses of the mammalian host. Further studies will help to determine whether ITPA activity is essential for parasite proliferation in vivo where the burden of deaminated (d)NTPs may be biologically significant and disrupt normal cell function.

## Methods

### Expression and purification of recombinant TbITPA protein

The ITPA coding sequence (Tb927.10.9990) was amplified from *T*. *brucei brucei* (Lister 427) genomic DNA using specific primers 5′-GCT AGC ATG ACT GAC GCC AGT AAA AC-3′ and 5′-GGA TCC CTA TTT GCT ATT TTC AAA ATG-3′. The PCR product was cloned into pET28a vector that allows protein expression in fusion to a histidine tag at the N-terminus (Novagen). Histidine-tagged TbITPA protein was purified on Ni^2+^-charged HiTrap Chelating HP columns (GE Healthcare) as described^51^. The protein-containing eluates were pooled, desalted using a PD-10 column (GE Healthcare) and applied to a Mono Q HR 5/5 column (GE Healthcare) at 1 mL min^−1^. Proteins were eluted with an increasing salt gradient generated using Buffer A (20 mM Tris–HCl, pH 8.0) and Buffer B (20 mM Tris–HCl, pH 8.0, 1 M NaCl). The fractions containing the TbITPA protein eluted at 80 mM NaCl approximately and were stored at − 80 °C in a buffer containing 20 mM Tris–HCl pH 8.0, 60 mM NaCl, 1 mM DTT and 15% glycerol. The purity was estimated on 12% SDS-PAGE.

The molecular mass determination of ITPA by gel filtration chromatography was made by comparing the ratio of Ve/Vo for the protein in question to the Ve/Vo (Ve is the elution volume and Vo is the void volume) of protein standards of known molecular mass (Sigma-Aldrich). The Vo of a given column is based on the volume of effluent required for the elution of a large molecule such as blue dextran. Plotting the logarithms of the known molecular masses of protein standards versus their respective Ve/Vo values produces a linear calibration curve.

### Enzymatic assays and determination of kinetic parameters

For a quantitative analysis of the ITPA reaction products, ribonucleotides (10–400 μmol) were incubated in reaction buffer [20 mM Tris–HCl pH 7.5, 5 mM MgCl_2_, 0.1 mg mL^−1^ BSA, 1 mM DTT] with concentrations of the enzyme ranging between 0.5 and 10 nM at 25 °C for 5 min (100 μL final volume). Reactions were stopped with EDTA and samples separated on a MonoQ 5/50 GL column (GE Healthcare) using an AKTAbasic chromatography system (GE Healthcare). The column was equilibrated in 5 mM sodium phosphate (pH 7.0) and 50 mM NaCl. Reaction products were eluted in a salt gradient from 50 to 350 mM of NaCl at a flow rate of 0.5 mL min^−1^ and detected at a 254 nm wavelength. All nucleotides used in the assays were obtained from Jena Bioscience.

Kinetic analysis was performed using the enzymatic assay and reaction conditions described above. Reaction rates were measured in the linear range of the reaction. In all cases, seven independent data points from three independent experiments were used. To determine *K*_m_, *k*_cat_ and *K*_i_ values, the data were fitted to a non-linear kinetic model for substrate inhibition and analyzed with GraphPad Prism 5 software. The *K*_m_ and *k*_cat_ values were used to calculate the catalytic efficiency for different ribonucleotide substrates (*k*_cat_/*K*_m_).

### Antibody generation

To obtain a polyclonal anti-TbITPA antibody, a rabbit was immunized using soluble purified recombinant TbITPA. Before injecting the antigen into the rabbit, 500 μg of pure protein diluted in PBS was mixed with Freund’s adjuvant (1:1 ratio). Antibody-containing serum was collected and affinity-purified using pure recombinant protein coupled to Affi-Gel 10 resin (BioRad) following the manufacturer’s instructions.

### Trypanosome growth and generation of transgenic cell lines

All cell lines used in this work derive from the *T. brucei brucei* single marker bloodstream form (BSF)^52^. Bloodstream cells were cultured at 37 °C and 5% CO_2_ in HMI-9 medium supplemented with 10% (v/v) of fetal bovine serum (FBS). Trypanosomes were transfected by electroporation in Cytomix medium for bloodstream cells, as previously described^52,53^. Clones were selected with appropriate selection drugs at the following concentrations: puromycin (Sigma-Aldrich), 0.1 μg mL^−1^; hygromycin (Sigma-Aldrich), 5 μg mL^−1^ and blasticidin (Invitrogen), 1 μg mL^−1^. Myc-ITPA expression was induced using 1 μg mL^−1^ of doxycycline (Sigma-Aldrich).

To generate knock-out and overexpression constructs, DNA fragments were amplified by PCR using *T*. *brucei* 427 genomic DNA as template and primers with sequences obtained from TriTrypDB (Tb927.10.9990). To generate *Tb*ITPA knockout cells, both alleles were replaced by genetic markers that provide resistance to hygromycin and blasticidin flanked by approximately 500 base pairs of the 3′ and 5′ *Tb*ITPA UTRs. The 5′-UTR was amplified with primers 5′-GCG GCC GCG AAC GAG ATG TTC CTT AGT CTA GTC GGG G-3′ (forward) and 5′-CTC GAG TAT ATT CAA AGA CTT ACG GAA GTA GTC TCC GTT C-3′ (reverse), and the 3′-UTR with primers 5′-GCA TGC GGT GCT GTT GTT GGT GTG GTG ATC GAC-3′ (forward) and 5′-GCT AGC GCT CCA CTC CAA TAA GTA CGA GCA CC-3′ (reverse). NotI restriction site was included (underlined) to allow the linearization of the plasmid. To overexpress a construct of *Tb*ITPA fused to a N-terminal myc tag, the coding sequence was amplified using primers 5′-ATT AAT ATG ACT GAC GCC AGT AAA AC-3′ and 5′-GGA TCC CTA TTT GCT ATT TTC AAA ATG-3′ including AseI and BamHI restriction sites. The PCR product was cloned in frame with the myc sequence at the NdeI and BamHI sites of a pGR23b-derived expression vector^54^. Knockout and myc-ITPA transfected cells were analyzed by PCR and Western blot analysis.

### Proliferation assays

Log-phase parasites at 10^4^ cells mL^−1^ were exposed to increasing concentrations of inhibitor or nucleoside analog for 24 h at 37 °C. Cells were counted using a Z1 Coulter counter and experiments for each compound were performed at least three times, in duplicate. Initial assays were carried out in HMI-9 medium which contains hypoxanthine (1 mM). Where indicated, hypoxanthine (1 mM) was substituted with guanine (100 μM) or hypoxanthine and guanine (100 μM both). Mycophenolic acid and ribavirin were purchased from Sigma-Aldrich.

### Subcellular localization studies

Intracellular localization of TbITPA was studied by immunofluorescence analysis. Briefly, log-phase parasites were harvested and incubated with MitoTracker Red CMX Ros (10 nM) (Invitrogen) for 15 min at 37 °C before being fixed in 4% PFA in wash solution [1X PBS, 0.2% Tween-20] on a poly-l-lysine coated slide for 20 min. Fixed parasites were washed twice in wash solution and permeabilized and blocked with 1% of IGEPAL (Sigma-Aldrich) in blocking solution [1X PBS, 0.2% Tween-20, 1% blocking reagent (Roche)] during 75 min. Immunostaining was performed by incubation with either rabbit polyclonal anti-TbITPA (1:100) or mouse monoclonal anti-c-myc (Sigma-Aldrich, 1:40) primary antibodies in blocking solution for 1 h. Subsequently, cells were labeled with Alexa Fluor 488 goat anti-rabbit IgG (Sigma-Aldrich, 1:40) or anti-mouse IgG (Sigma-Aldrich, 1:40) were used as secondary antibody, respectively. After washing, coverslips were dehydrated in methanol for 1 min and stained and mounted with ProLong Gold Antifade Reagent with DAPI (Life Technologies).

Vertical stacks of 30–40 slices (0.2 μm steps) were captured using an Olympus wide-field microscope and Cell R IX81 software. Images were deconvolved and pseudocolored with Huygens Essential software (version 3.3; Scientific Volume Imaging). All images were analyzed using Fiji/ImageJ software (version 1.5e; ImageJ)^55^. Degree of colocalization was determined using Pearson’s correlation coefficient and Manders’ overlap coefficient. Both coefficients were calculated with JACoP plugin^56^ and given values are the mean of at least 20 cells.

### Ribavirin triphosphate detection in cell extracts

Cells were washed twice with ice-cold 1X PBS and resuspended in ice-cold dH_2_O (50 μL). Samples were treated with 0.4 M perchloric acid (50 μL) and incubated 5 min on ice before centrifugation at 16,000*g* for 10 min at 4 °C. Immediately after rNTPs extraction, 100 μL of sample were mixed with 100 μL of 0.5 M ammonium phosphate buffer (pH 3.4) and loaded into a Partisphere 5-µm SAX column. Discrimination of rNTPs was performed on isocratic conditions for loading and elution with 0.5 M ammonium phosphate buffer, pH 3.4. Known standards were loaded in order to properly identify each nucleotide.

### Statistics

Student’s *t*-test was used to compare different sets of data. Two-way ANOVA was used to analyse normally distributed data, followed by Dunnet’s post-hoc test. Statistics were calculated with IBM SPSS Statistics or GraphPad Prism 5. Results are expressed as means ± SD of at least three independent experiments. *P < 0.05, **P < 0.01, ***P < 0.001.

## Supplementary Information

Supplementary Figures.

## Data Availability

The authors declare that the data supporting the findings of this study are available within the article and its supplementary information files.
