# Supplementary material for: Inosine triphosphate pyrophosphatase from Trypanosoma brucei cleanses cytosolic pools from deaminated nucleotides
Source: Sci Rep. 2022 Apr 18;12:6408. doi: 10.1038/s41598-022-10149-4 (PMC9016069; doi:10.1038/s41598-022-10149-4)
Supplement: Supplementary file 1 — Supplementary Figures. [file 41598_2022_10149_MOESM1_ESM.pdf]

Inosine triphosphate pyrophosphatase from *Trypanosoma brucei* cleanses cytosolic pools from deaminated nucleotides

Antonio E. Vidal, Miriam Yagüe-Capilla, Blanca Martínez-Arribas, Daniel García-Caballero, Luis M. Ruiz-Pérez, and Dolores González-Pacanowska.

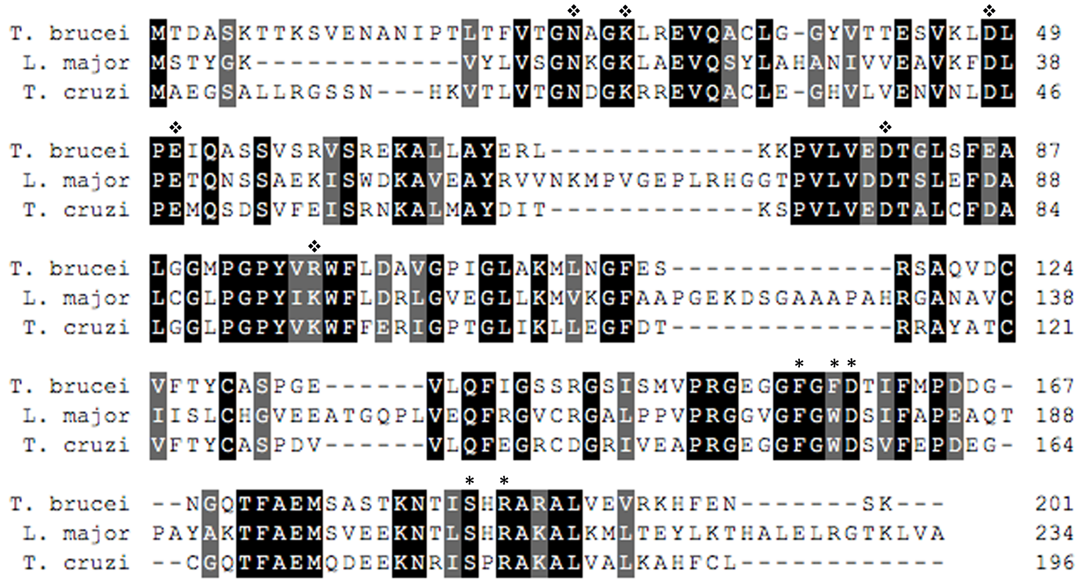

Figure S1. Sequence alignment of kinetoplastid ITPAs. Multiple sequence alignment was generated using T-Coffee and the final format was obtained using Multiple Align Show. The amino acids involved in substrate selectivity (*) and catalysis (❖) are indicated. Protein sequences were retrieved from TriTrypDB: *T. brucei*, *Trypanosoma brucei* (Tb927.10.9990); *L. major*, *Leishmania major* (LmjF.36.4630); *T. cruzi*, *Trypanosoma cruzi* (TcCLB.504103.6).

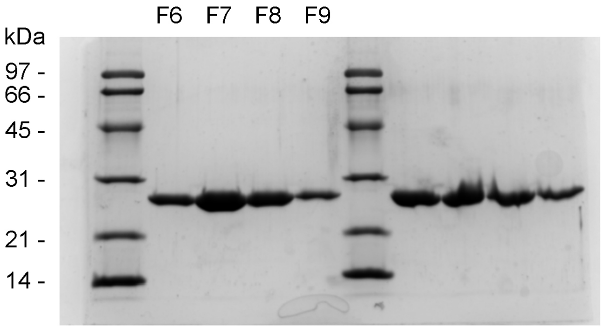

Figure S2. Purified protein was separated on SDS-PAGE gel and stained with Coomassie. M, molecular weight markers; F6-F9, fractions containing purified TbITPA were obtained after affinity and anion exchange chromatography. On the right side of the gel, fractions obtained from affinity chromatography before being pooled and desalted.

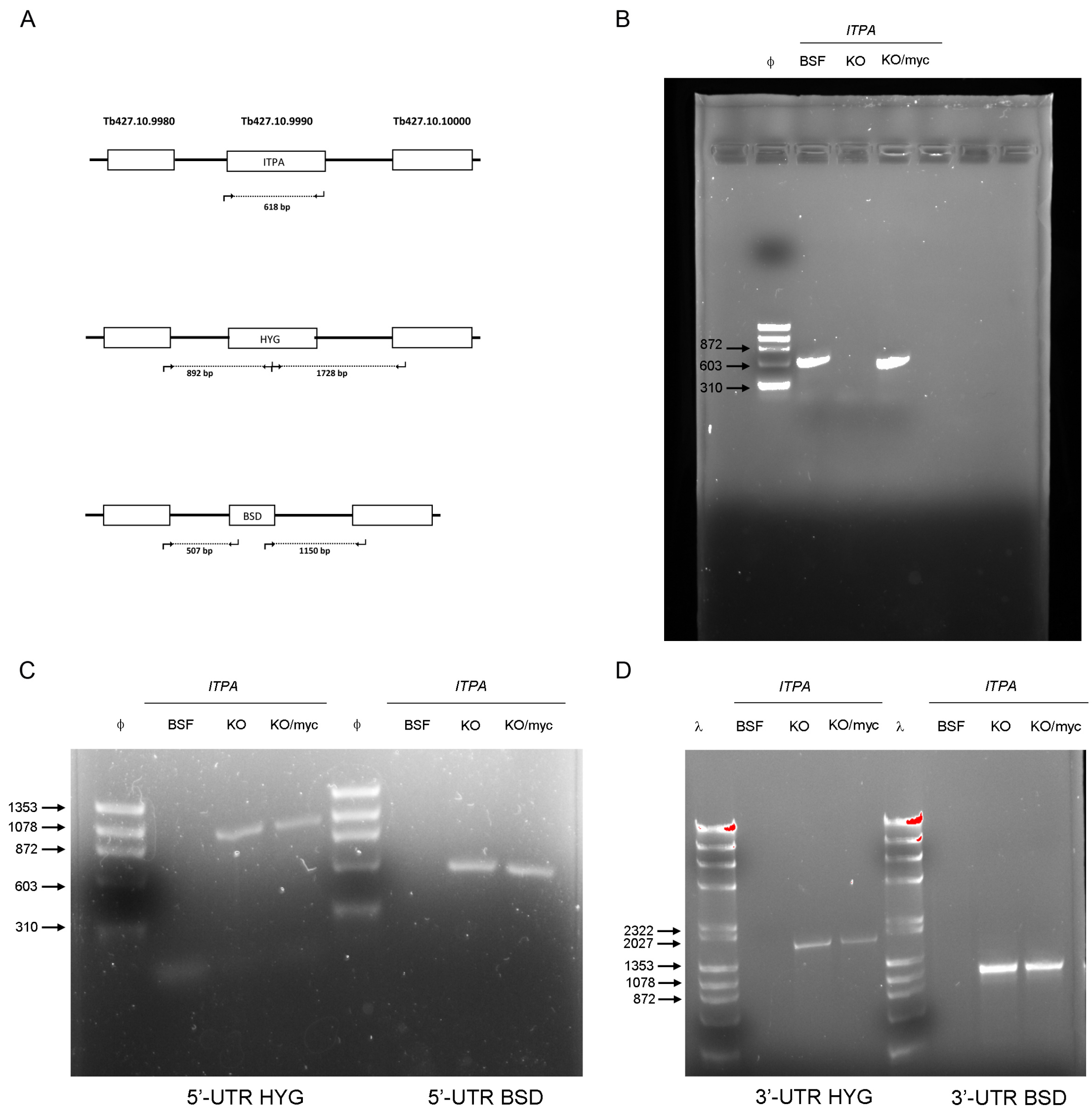

Figure S3. ITPA disruption by double allele replacement. A) Schematic drawing showing the PCR assays performed to confirm the replacement of the ITPA locus by hygromycin phosphotransferase (HYG) and blasticidin S deaminase (BSD). Amplification assays included in the figure were carried out with genomic DNA from clones selected for further study. The position of the amplification primers is represented by arrows. The expected sizes of the PCR products are also indicated. (B) Detection of the ITPA ORF (618 bp) by PCR amplification with specific primers. (C) Integration of the HYG and BSD markers was monitored by PCR using primers complementary to sequences of loci located upstream of the inactivation cassette in combination with marker-specific primers. (D) PCR assays using primers complementary to sequences of loci located downstream of the inactivation cassette in combination with marker-specific primers. λ, ϕ, DNA ladders.

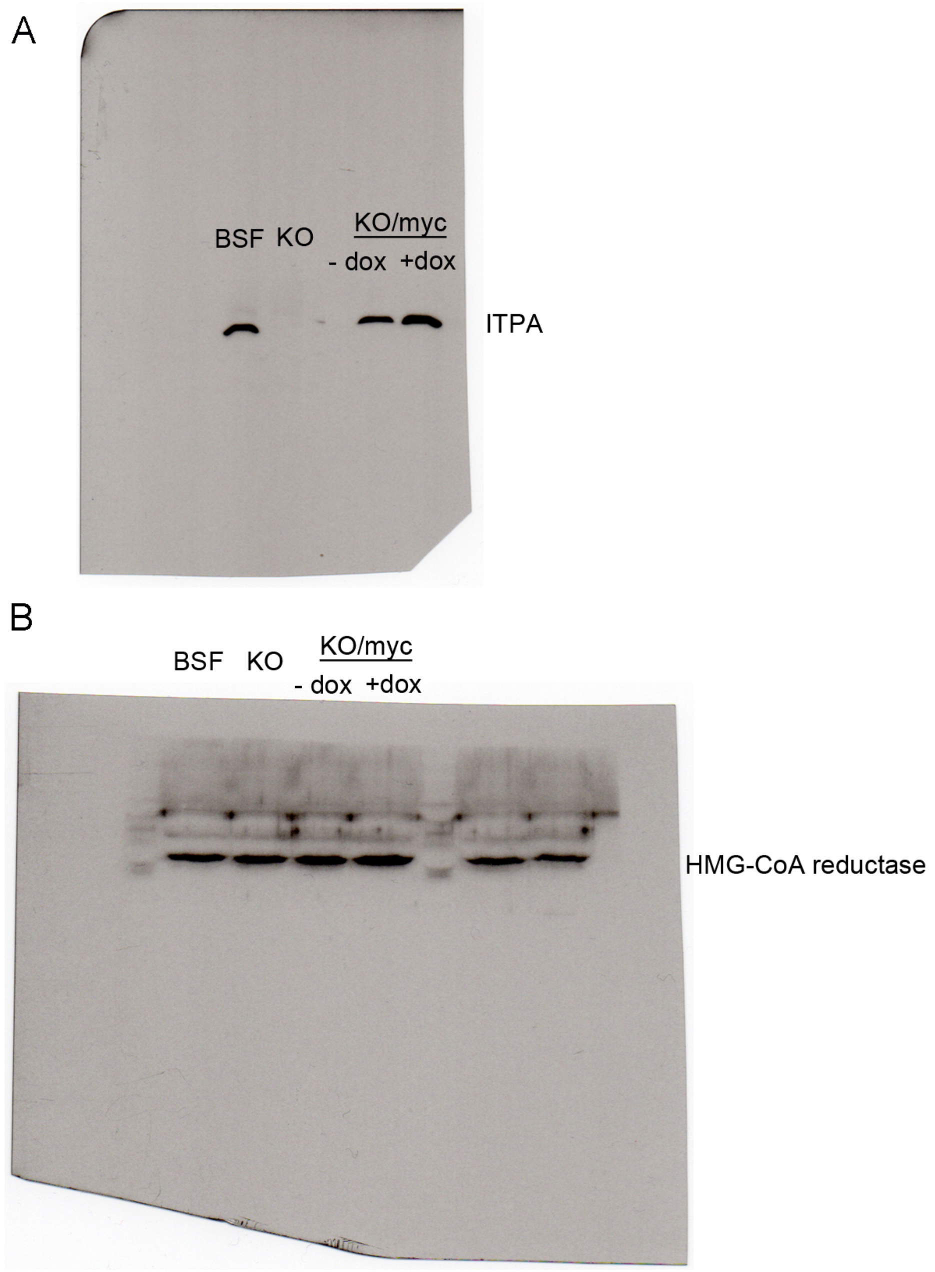

Figure S4. Full size images of western blots presented in figure 3 of the manuscript. (A) Western blot showing ITPA protein levels in whole cell extracts from $5x10^6$ parasites. Cell lines analyzed include: wild-type bloodstream form (BSF), ITPA-KO cells (KO) or ITPA-KO cells harboring an ectopic inducible copy of a myc-ITPA fusion gene (KO/myc) in the absence (-dox) or presence (+dox) of the inductor. (B) As loading control, HMG-CoA reductase protein was measured in the three cell lines in a separate western blot.

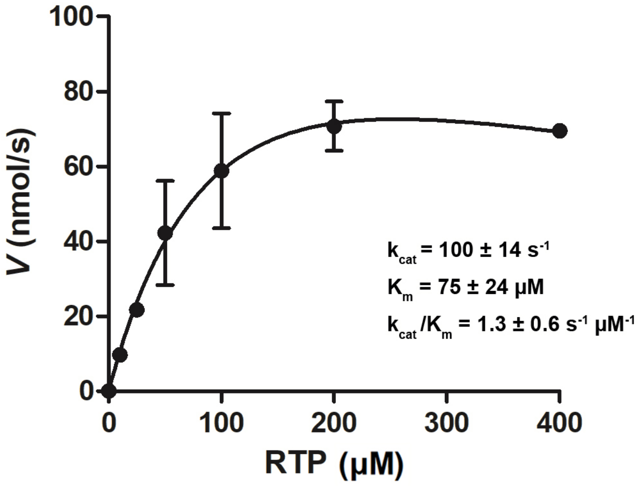

Figure S5. Michaelis-Menten plot for the hydrolysis of ribavirin triphosphate by TbITPA. Substrate saturation curve obtained for ribavirin triphosphate (RTP). Each data point is the mean (±SD) of at least three independent determinations. Reactions were performed and analyzed as described in Materials and Methods. The data were fitted to a Michaelis-Menten kinetic model and kinetic parameters determined with GraphPad Prism 5 software.
